# The complete mitochondrial genome of *Lycodon ruhstrati* (Serpentes: Colubridae)

**DOI:** 10.1080/23802359.2019.1623730

**Published:** 2019-07-10

**Authors:** Yan-An Gong, Li-Fang Peng, Shun-Qing Lu, Song Huang

**Affiliations:** College of Life and Environment Sciences, Huangshan University, Huangshan, China

**Keywords:** *Lycodon ruhstrati*, mitogenome, phylogeny

## Abstract

The complete mitochondrial genome sequence of *Lycodon ruhstrati* was determined by shotgun sequencing. The total genome size is 17,168 bp, and contains 13 protein-coding genes, 22 tRNA genes, 2 ribosome RNA genes and 2 control regions (D-loops). Most of the genes of *L*. *ruhstrati* were distributed on the H-strand, except for the ND6 subunit gene and eight tRNA genes which were encoded on the L-strand. The phylogenetic tree of *L*. *ruhstrati* and 12 other closely related species was reconstructed. The mitogenome sequence presented here will be useful to study the evolutionary relationships and genetic diversity of *L*. *ruhstrati*.

The genus *Lycodon* is one of the most diverse groups of lizards with more than15 known species (Cai et al. 2015). *Lycodon ruhstrati* (Fischer, 1886) was firstly reported as *Ophites ruhstrati* by Fischer from Taiwan province, China in 1886. This species is currently distributed in northeast of Vietnam and most area of China (Zhao et al. 1999; Wang et al. 2003; Wang and Zheng 2005; Chen et al. 2006; Zhao 2006; Guo et al. 2007; Vogel et al. 2009). In this research, we determined and described the mitogenome sequence of *L. ruhstrati* in order to obtain basic genetic information about this species.

During investigation of taxonomy of amphibians and reptiles in Zhejiang province in June of 2018, we sampled a female adult specimen of *L. ruhstrati* in Qilang mountain, Kaihua county. It was preserved and deposited in the Museum of Huangshan University (Voucher numbers: HSR18050). Fresh liver tissues were removed and immediately preserved in 95% ethanol. Total genomic DNA was extracted from muscle using a Qiagin DNEasy blood and tissue extraction kit (Qiagen Inc., Valencia, CA). The complete mitogenome sequence has been submitted to GenBank with accession number is MK867843.

The total genome sequence length of *L. ruhstrati* was sequenced to be 17,168 bp which consisted of 13 typical vertebrate protein-coding genes (PCGs), 22 transfer RNA (tRNA) genes, 2 ribosomal RNA (rRNA) genes and 2 control regions. Most of the mitochondrial genes are encoded on the H-strand except for the ND6 gene and eight tRNA genes, which are encoded on the L-strand. Among the mitochondrial protein coding genes, the ATP8 was the shortest, while the ND5 was the longest. The positions of RNA genes were predicted by the MITOS (Bernt et al. 2013), and the locations of protein-coding genes were identified by comparing with the homologous genes of other related species. The complete mitogenome has been obtained from shotgun sequencing. The base composition was 34.3% for A, 24.8% for T, 12.6% for G and 28.2% for C. The gene order, contents and base composition are identical to those found in typical vertebrates (Boore 1999; Sorenson et al. 1999).

The phylogenetic tree of *L. ruhstrati* was reconstructed based on the complete mtDNA sequences with other 12 related species from GenBank by MEGA 7.0 (Kumar et al. 2016) using Maximum-likelihood (ML) methods. The ML tree (Figure 1) was reconstructed in http://www.phylo.org/portal2/login!input.action. The phylogenetic analysis result was consistent with the previous research with a highly support. It indicated that our new determined mitogenome sequences could meet the demands and explain some evolution issues.

**Figure 1.**
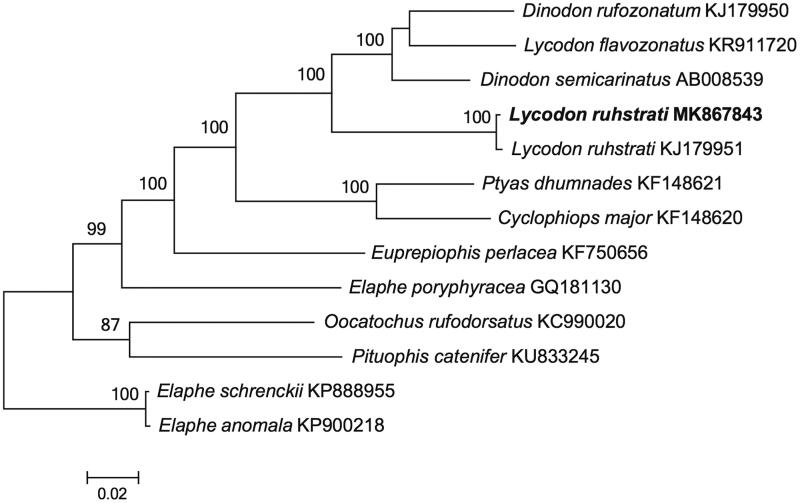
A maximum likelihood (ML) tree of Lycodon ruhstrati in this study and 12 related species was constructed based on the dataset of the whole mitochondrial genome by online tool RAxML. The numbers above the branch meant bootstrap value. Bold black branches highlighted the study species and corresponding phylogenetic classification.
